# The Role of Mesenchymal Stem Cells with Ascorbic Acid and N-Acetylcysteine on TNF-*α*, IL 1*β*, and NF-*κβ* Expressions in Acute Pancreatitis in Albino Rats

**DOI:** 10.1155/2021/6229460

**Published:** 2021-10-16

**Authors:** Dalia Abdelhafez, Elshimaa Aboelkomsan, Abir El Sadik, Noha Lasheen, Sara Ashur, Amal Elshimy, George N. B. Morcos

**Affiliations:** ^1^Department of Pathology, Faculty of Medicine, Fayoum University, Fayoum, Egypt; ^2^Department of Pathology, Faculty of Medicine, New Giza University, Cairo, Egypt; ^3^Department of Anatomy and Histology, College of Medicine, Qassim University, Saudi Arabia and Department of Anatomy and Embryology, Faculty of Medicine, Cairo University, Cairo, Egypt; ^4^Department of Physiology, Faculty of Medicine, Ain Shams and Galala Universities, Cairo, Egypt; ^5^Department of Biochemistry and Molecular Biology, Faculty of Medicine, Cairo University, Cairo, Egypt; ^6^Department of Medical Microbiology and Immunology, Faculty of Medicine, Cairo University, Cairo, Egypt; ^7^Department of Medical Biochemistry and Molecular Biology, Faculty of Medicine, Cairo University, and Department of Basic Medical Science, Faculty of Medicine, King Salman International University, Cairo, Egypt

## Abstract

Severe acute pancreatitis (SAP) is a necrotic pancreatic inflammation associated with high mortality rate (up to 70%). Bone marrow (BM) mesenchymal stem cells (MSCs) have been investigated in pancreatic cellular regeneration, but still their effects are controversial. Therefore, the present study is aimed at examining the enrichment of the stem cells with ascorbic acid (AA) and N-acetylcysteine (NAC) and explore their combined action on the expression of the inflammatory cytokines: interleukin 1*β* (IL 1*β*), tumor necrosis factor-*α* (TNF-*α*), and nuclear factor-*κβ* (NF-*κβ*). A total of twenty adult male *Sprague-Dawley* albino rats were divided into four groups: the control group, cerulein group (to induce acute pancreatitis), BM-MSCs group, and combined BM-MSCs with AA and NAC group. Homing and proliferation of stem cells were revealed by the appearance of PKH26-labelled BM-MSCs in the islets of Langerhans. AA and NAC combination with BM-MSCs (group IV) was demonstrated to affect the expression of the inflammatory cytokines: IL 1*β*, TNF-*α*, and NF-*κβ*. In addition, improvement of the biochemical and histological parameters is represented in increasing body weight, normal blood glucose, and insulin levels and regeneration of the islet cells. Immunohistochemical studies showed an increase in proliferating cell nuclear antigen **(**PCNA) and decrease in caspase-3 reactions, detected markedly in group IV, after the marked distortion of the classic pancreatic lobular architecture was induced by cerulein. It could be concluded that treatment with BM-MSCs combined with antioxidants could provide a promising therapy for acute pancreatitis and improve the degeneration, apoptosis, necrosis, and inflammatory processes of the islets of Langerhans. TNF-*α*, IL 1*β*, and NF-*κβ* are essential biomarkers for the evaluation of MSC regenerative effectiveness.

## 1. Introduction

Severe acute pancreatitis (SAP) is a serious acute inflammation of the pancreas. Approximately five to ten percent of SAP patients develop severe parenchymal necrosis of the pancreas [1]. Fulminant or subfulminant pancreatitis is usually associated with systemic inflammatory response syndrome (SIRS), multiple organ dysfunction syndrome (MODS), and a high mortality rate (up to 70%) [2, 3]. Multiple inflammatory cytokines are involved in the pathogenesis of SAP, including proinflammatory cytokines such as interleukin-1 (IL-1), IL-6, and tumor necrosis factor-*α* (TNF-*α*) and anti-inflammatory cytokines such as IL-10 [4, 5]. Nuclear factor-*κβ* (NF-*κβ*) is one of the Rel protein family of transcription factors that regulate the expression of genes related to cellular stress and cytokine production [6], which is comparable to the expression of the Wnt/*β*-catenin pathway in chronic mild stress as a potential regulator of microglia-mediated neuroinflammation [7]. This factor is kept silent in the cytoplasm through the inhibitor of NF-*κβ* (I*κβα*). Slow degradation of I*κβα* has been reported in induced acute pancreatitis (AP), leading to NF-*κβ* activation. NF-*κβ* activation has been demonstrated in induced AP in response to the oxidative stress within the acinar cells, which was associated with upregulation of TNF-*α* [8].

Experimental therapeutic attempts for the inhibition of TNF-*α* production or administration of anti-TNF-*α* have been associated with reduced tissue damage and reduced mortality rate of SAP in animal models [9–12]. Although the therapeutic role of other cytokines, such as IL-1*β* and NF-*κβ*, is controversial, some studies observed that IL-1*β*, prostaglandin E2 (PGE2), and NF-*κβ* signaling allowed inhibition of SIRS and treatment of experimental SAP [13–16]. Another study proposed that NF-*κβ* activation resulted in increased apoptosis and necrotizing pancreatitis associated with MODS [17].

In previous experimental studies, injection of taurocholic acid was used to induce the SAP model, by induction of acute biliary pancreatitis. However, the difficulties of surgery on small animals held up taurocholic acid usage [18]. Therefore, researchers used cerulein to produce SAP in the experimental animals. Cerulein was proven to induce rapidly disseminated pancreatic injury very similar to that of human pancreatitis [19, 20]. Treatment of AP is still a challenge as there are no satisfactory therapeutic measures available to promote pancreatic regeneration.

Bone marrow (BM) mesenchymal stem cells (MSCs) have been reported to allow tissue regeneration and release soluble factors which modulate the immune response, in addition to their high differentiation ability to many other lineages. MSCs were previously proven to differentiate and allow cellular regeneration of many tissues such as the gastrointestinal cells, nerve cells, cardiomyocytes, cartilage, and liver tissue. Jiang et al. [21] and Takahashi et al. [22] have reported that administration of BM-MSCs alleviated SAP in rats. Therefore, stem cell biology and BM-MSC transplantation are becoming a field of interest for many therapeutic studies [23].

N-acetylcysteine (NAC) is a thiol compound. It is considered a synthetic precursor of the glutathione. NAC is a nucleophile that can bind with the reactive metabolites and increase the activity of the glutathione transferase enzyme [24]. NAC has potent antioxidant and anti-inflammatory properties [25]. Another antioxidant is ascorbic acid (AA) or vitamin C which is one of the essential water-soluble vitamins. AA is needed for many physiological functions in the human body mainly through inhibition of oxidative stress [26]. Several human studies have demonstrated the reduction of the harmful oxidation in the stomach and blood vessels through the antioxidant effect of vitamin C. The role of vitamin C in the improvement of biochemical parameters, including blood glucose level and insulin secretion, has been reported also in albino rats [27]. It remains uncertain, whether the protective antioxidant effect of NAC and AA can enhance the regenerative efficacy of BM-MSC transplantation in SAP. Currently, there are no definite efficient therapeutic measures for SAP. The high differentiation potential of BM-MSCs makes them a promising therapeutic option. In the present study, the authors investigated the therapeutic feasibility of BM-MSCs individually and in combination with ascorbic acid and N-acetylcysteine antioxidants on cerulein-induced SAP in albino rats.

## 2. Material and Methods

### 2.1. Isolation and Culture of MSCs

Fluorescent-labelled bone marrow MSCs were prepared at the Biochemistry and Molecular Biology Unit, Faculty Medicine, Cairo University. Bone marrow stromal cells were harvested by flushing the femurs and tibiae of albino rats with Dulbecco's modified Eagle's medium (DMEM) (Sigma, USA, D5796) with the addition of 10% fetal bovine serum (FBS) (Sigma, USA, F6178). The cells were layered in a ratio of 2 : 1 over Ficoll-Hypaque (Sigma, USA, F8016) in sterile conical tubes and then centrifuged. Aspiration of the mononuclear cells was done, and they were suspended in complete culture medium supplemented with 1% penicillin-streptomycin (Sigma, USA, P4333) and incubated at 37°C in 5% humidified CO_2_ for 14 days with change of the media every 4 days. The cultures were washed twice with phosphate buffer saline (PBS) (Sigma, USA, P5493) at 80% confluence indicated by development of large colonies. The cells were trypsinzed with 0.25% trypsin (Sigma, USA, T1426) in 1 ml ethylene diamine tetra acetate (EDTA) (Sigma, USA, E6758) for 5 minutes at 37°C and centrifuged at 2400 rpm for 20 minutes. The cell pellets were suspended with serum supplemented medium and incubated in 25 cm^2^ culture flasks forming the first passage cultures [28, 29].

### 2.2. Immunophenotyping of the MSCs

The bone marrow MSCs were washed and suspended in PBS. CD29 (Sigma, USA, SAB4501582) and CD45 (Sigma, USA, OX-1 84112004) monoclonal antibodies were added to the cells and kept for 1 hour in 4°C. Incubation of the cells with anti-mouse immunoglobulin G fluorescein-conjugated secondary antibody (Millipore Corp., Temecula, CA) was implemented for 45 minutes on ice. Cell suspensions were washed twice and then analyzed on a FACSCalibur flow cytometer [30, 31].

### 2.3. Labelling of the MSCs with PKH26

MSCs were labelled with fluorescent PKH26 (Sigma, USA, MINI26) according to the manufacturer's recommendations [32]. Detection of cell viability was done by adding 1 : 1 ratio of cell suspension and 0.4% trypan blue stain. Viable cells appeared shiny without staining under the phase contrast microscope [33].

### 2.4. Animals

A total of twenty adult male *Sprague*-*Dawley* albino rats weighing 200-250 g each and 4-6 months old were used in the current study. They were locally bred at the animal house at the Faculty of Medicine, Cairo University, Egypt. The animals were given two weeks' acclimatization period before starting the experiment. The animals had access to food and water ad libitum and were housed at room temperature. The experiment proposal was approved by the Ethics Committee, College of Medicine, Cairo University. The rats were treated in accordance with the international guidelines for the care and use of laboratory animals including the way of animal treatment, anesthesia, methodology of the collection of the MSCs from the animal's bone marrow, and their use in the experimental research. Minimal animal sufferings were ensured.

### 2.5. Experimental Design

The animals were divided randomly into four groups of five rats each as follows.

#### 2.5.1. Group I (Control Group)

The rats received two intraperitoneal injections of 0.9% saline at two-hour intervals, one systemic injection (through the caudal vein) of 0.5 ml phosphate-buffered saline (PBS) (P5493, Sigma, USA) without MSCs and 1 ml saline orally with gastric gavage once daily for 30 days.

#### 2.5.2. Group II (Cerulein-Treated Group)

Two intraperitoneal injections of 100 *μ*g/kg body weight of cerulein (Sigma-Aldrich Co., Taufkirchen, Germany) was applied to the five rats of the group at a two-hour interval; each injection contained 50% of the dose [34].

#### 2.5.3. Group III (Cerulein and MSCs-Treated Group)

The rats received one systemic injection (through the caudal vein) of MSCs (1 × 10^6^) diluted in 0.5 ml of PBS just before cerulein injection at the same dose of group II [29, 35].

#### 2.5.4. Group IV (Cerulein, MSCs, and Antioxidant Mixture-Treated Group)

The rats received 100 mg/kg body weight of L-ascorbic acid [36] and a similar dose of N-acetylcysteine (SEDICO Pharmaceutical Company, 6th of October, Egypt) [37], orally with gastric gavage in 1 ml saline vehicle per dose, once daily for 30 days, one systemic injection (through the caudal vein) of MSCs (1 × 10^6^) diluted in 0.5 ml of PBS and cerulein at the same dose of group II.

At the end of the experiment, on the 30^th^ day, blood samples were collected from the retroorbital plexus using capillary glass tubes. The rats were weighted and sacrificed by intraperitoneal injection of overdose of pentobarbital: 40 mg/kg body weight. The pancreas of each animal was dissected. Specimens were fixed for light microscopic, fluorescent, and immunohistochemical studies. Other specimens were prepared directly for gene expression studies [38].

### 2.6. Biochemical Studies

#### 2.6.1. Blood Glucose Level

Three days after injection with cerulein, random blood glucose levels were detected using a glucometer (ACON Laboratories, Inc., USA). The rats were tested for hyperglycemia and diagnosed diabetic when the random blood glucose level became higher than 220 mg/dl [39]. Blood glucose levels were measured also at the end of the experiment.

#### 2.6.2. Fasting Serum Insulin

Fasting serum insulin level was determined using the Ultra-Sensitive Mouse Insulin Enzyme-Linked Immunosorbent Assay (ELISA) Kit (Crystal Chem) from the blood samples collected just before rat scarification [40].

### 2.7. Detection of Studied Genes by Quantitative Real-Time Polymerase Chain Reaction (QRT-PCR)

The specimens obtained from the pancreas of all rats (0.2 mg) were homogenized in PBS, pH 7.4 using tissue Lyzer (Qiagen; Hilden, Germany). The homogenate was centrifuged at 8000 xg for 20 minutes; then, the supernatant was used for total RNA extraction. Total RNA was extracted using the RNeasy Mini Kit; cat no: 217004 (Qiagen, Hilden, Germany) according to the manufacturer's protocol. cDNA was synthesized by reverse transcription reaction using QuantiTect Reverse Transcription Kit; cat no: 205311; (Qiagen, Hilden, Germany). The gene expression for the tumor necrosis factor alpha (TNF-*α*), interleukin 1 beta (IL-1*β*), and nuclear factor kappa *β* (NF-*κβ*) levels was amplified from cDNA using the QuantiTect SYBR Green PCR Kit cat no: 204141 (Qiagen, Germany) and the QuantiTect primer assays cat no: 249900 ((Rn_Tnfrsf1a_1_SG QuantiTect Primer Assay, ID QT00388346), (Rn_Il1b_1_SG QuantiTect Primer Assay, ID QT00181657) and (Rn_Nfkb2_1_SG; ID: QT00396823)), , respectively. The ACTB primer sequence was used as a housekeeper gene. All samples were analyzed using the 5plex Rotor-Gene PCR Analyzer (Qiagen, Germany). The 2^*ΔΔ*Ct^ method was conducted for the analysis of gene expression levels, using ACTB as an endogenous reference control for normalization purposes [41].

### 2.8. Light Microscopic Study

The dissected pancreatic specimens were fixed in 10% formaldehyde solution, processed and embedded to obtain paraffin blocks, and cut at 5 *μ*m thickness sections. The sections were deparaffinised in xylol solution then rehydrated in 100%, 95%, and then 70% alcohol and washed in distilled water. Sections were prepared for fluorescent study, and others were subjected for the following examinations.

### 2.9. Hematoxylin and Eosin (H&E) Stain

Half of the sections, prepared from the pancreatic specimens for light microscopic studies, were stained with hematoxylin for ten minutes. The basophilic structures of the cytoplasm and the nuclei were stained with blue color. Then, the sections were stained in one percent aqueous eosin for three minutes. The acidophilic structures of the cytoplasm were stained with red color. The sections were dehydrated in alcohol (70%, 90% then 100%) then cleared by xylene. The slides were removed from xylol and mounted in Canada balsam and put on the cover slip [42].

### 2.10. Immunohistochemical Reaction

The rest of the pancreatic specimens prepared for light microscopic studies were cut at 5 *μ*m thickness and then collected on poly-L-lysine-coated slides. The sections were deparaffinised into two changes of xylene and rehydrated through graded washes of ethanol in water and finally rinsed in pure water. Then, they were treated with 0.9% hydrogen peroxide in absolute methanol for 10 min. Antigen retrieval was achieved by heating the sections in 10 mm sodium citrate buffer, in a water bath at 95°C for 30 minutes. The sections were rinsed twice in PBS Tween 20 for 2 minutes. Then, they were blocked with 5% normal mice serum for 30 minutes at room temperature. Incubation with the following primary antibodies was performed for 30 minutes:

1. Proliferating cell nuclear antigen (PCNA): this antigen is a cofactor of DNA polymerase-*δ* which is essential for DNA replication, DNA repair, and chromatin remodelling. It was detected by rabbit polyclonal IgG (FL-261; catalog number SC-7907, 200 *μ*g/ml, dilution 1 : 50, Santa Cruz Biotechnology, USA). Brown discoloration of the nuclei, in the proliferating cells, indicates positive reaction of nuclear regeneration [43]
2. Caspase-3 antibody: caspase-3 is an essential mediator of programmed cell death: apoptosis. Anti-caspase-3 mouse monoclonal primary antibody (Dako Company, Cairo, Egypt; catalog no. IMG-144A at a dilution 1/200) was used [44]. The slides were rinsed in PBS, incubated with 2 drops of biotinylated secondary antibody for each section for 20 minutes, and then rinsed with PBS. Substrate chromagen (DAB) mixture was applied for 5 minutes then rinsed with distilled water. The slides were stained with hematoxylin and then dehydrated and mounted. Brown discoloration of the cytoplasm indicates positive reaction of the apoptotic cells [45].

### 2.11. Histomorphometric Measurements

Ten nonoverlapping fields, randomly chosen per sections stained for immunohistochemical reactions, at a magnification of 400 were examined by an independent observer, using Leica LAS, V3.8 image analyzer computer system (Switzerland). The image analyzer was calibrated automatically to convert the measurement units (pixels) into micrometer units. The area percent of positive immune reaction for PCNA- and caspase-3-stained sections was measured. The area percent represented the areas of the positive reaction, masked by a binary blue color to the area bounded within a standard measuring frame (7286.783 *μ*m^2^) [46].

### 2.12. Statistical Analysis

All the measurements were expressed as mean and standard deviation (±SD) and subjected to statistical analysis using “SPSS 22” (SPSS, Inc., Chicago, Illinois, USA) software. Analysis of variance using one-way (ANOVA) and post-hoc tests were utilized for comparison between quantitative variables. Results were considered significant when the *p* value was less than 0.05 [47].

## 3. Results

### 3.1. Biochemical Results

#### 3.1.1. Body Weight

The mean body weight of the rats of group II (cerulein-treated group) was significantly decreased (43.6%) compared with the control group (group I). However, in group III (cerulein+MSCs-treated group), the body weight was significantly decreased (66.79%) compared with the control group (group I) and significantly increased compared with group II (cerulein-treated group). In addition, it was significantly decreased in group IV (cerulein+antioxidants+MSCs-treated group) compared with the control group (82.33%) and significantly increased compared with both group II (cerulein-treated group) and group III (cerulein+MSCs-treated group) (Table 1).

#### 3.1.2. Fasting Blood Glucose

The mean fasting blood glucose of the rats of group II (cerulein-treated group) was significantly increased compared with the control group (group I). However, in group III (cerulein+MSCs-treated group), the fasting blood glucose was significantly increased compared with the control group and significantly decreased compared with group II (cerulein-treated group). In addition, it was significantly increased in group IV (cerulein+antioxidants+MSCs-treated group) compared with the control group and significantly decreased compared with group II (cerulein-treated group) and group III (cerulein+MSCs-treated group) (Table 1).

#### 3.1.3. Fasting Serum Insulin

The mean fasting serum insulin of the rats of group II (cerulein-treated group) was significantly decreased compared with the control group (group I). However, in group III (cerulein+MSCs-treated group), it was significantly decreased compared with the control group and significantly increased compared with group II (cerulein-treated group). In group IV (cerulein+antioxidants+MSCs-treated group), it was significantly increased compared with only group II (cerulein-treated group) (Table 1).

### 3.2. Real-Time PCR for IL-1*β*, TNF-*α*, and NF-*κβ* Gene Expressions

The mean of the real-time PCR for IL-1*β* gene expression of the rats of group II (cerulein-treated group) was significantly increased compared with the control group (group I). However, in group III (cerulein+MSCs-treated group) and group IV (cerulein+antioxidants+MSCs-treated group), it was significantly increased compared with the control group and significantly decreased compared with group II (cerulein-treated group). The mean of the real-time PCR for TNF-*α* gene expression of the rats of group II (cerulein-treated group) was significantly increased compared with the control group (group I). In addition, in group III (cerulein+MSCs-treated group) and group IV (cerulein+antioxidants+MSCs-treated group), it was significantly decreased compared with group II (cerulein-treated group). The mean of the real-time PCR for the NF-*κβ* gene expression of the rats of group II (cerulein-treated group) was significantly increased compared with the control group (group I). However, in group III (cerulein+MSCs-treated group), it was significantly increased compared with the control group and significantly decreased compared with group II (cerulein-treated group). In addition, it was significantly increased in group IV (cerulein+antioxidants+MSCs-treated group) compared with the control group and significantly decreased compared with group II (cerulein-treated group) and group III (cerulein+MSCs-treated group) (Table 2).

### 3.3. Light Microscopic Results

#### 3.3.1. PKH26 Fluorescence Stain

**Figure 1:**
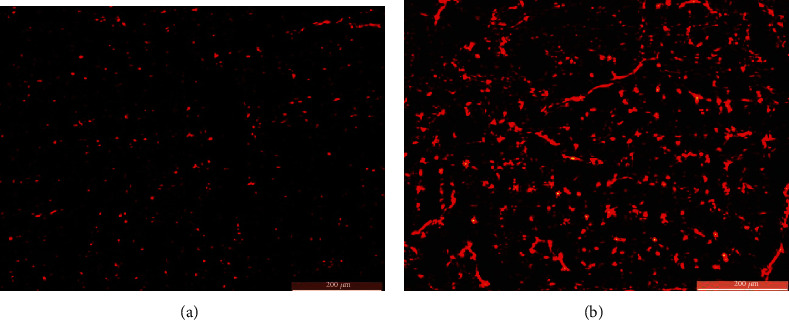
Photomicrographs of sections of the pancreatic specimens of the rats. (a) Group III showing homing of the PKH26-labelled masses of MSCs inside the pancreatic tissue. (b) Group IV showing increased density of homing and proliferation of the PKH26-labelled masses of MSCs inside the pancreatic tissue (PKH26 ×100).

MSCs with the cerulein-treated group (group III) showed homing of PKH26-labelled red fluorescent cell masses within the pancreatic tissue. While the specimens of the rats of group IV (MSCs, antioxidant mixture, and cerulein-treated group) revealed increase density of homing and proliferation of the PKH26-labelled masses of MSCs inside the pancreatic tissue (Figure 1).

#### 3.3.2. Hematoxylin and Eosin (H&E) Stain

**Figure 2:**
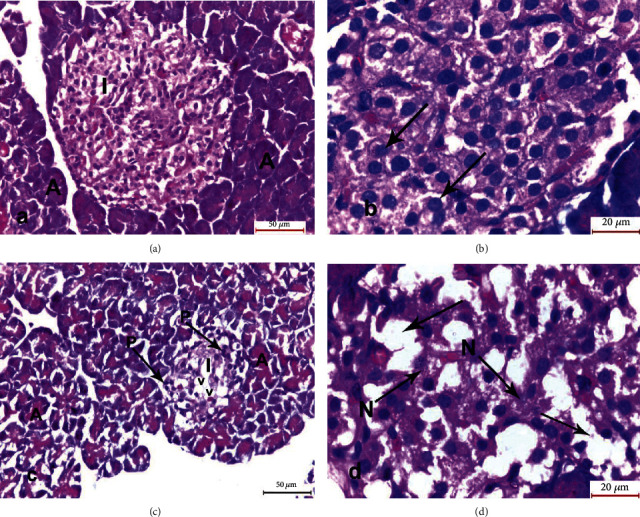
Photomicrographs of sections of the pancreatic specimens of the rats. (a) Group I showing normal architecture of the islet of Langerhans (I). It appears lightly stained, well defined with regular outline. It contains a large number of islet cells. The islet is surrounded by closely packed serous acini (A) with regular outline, rounded nuclei and dark cytoplasm. (b) Higher magnification of the islet of Langerhans of group I showing large number of islets cells having vesicular nuclei with prominent nucleoli (arrows). (c) Group II showing reduced dimension of islet of Langerhans (I) with ill-defined border. Fewer islet cells with pyknotic nuclei (P) and intracytoplasmic vacuolations (v) and distorted acini with degenerated cells (A) appeared. (d) Higher magnification of group II showed necrotic cells (N) with many empty spaces (arrows) (H&E: a, c ×400; b, d ×1000).

The sections of the pancreatic specimens of the rats of the control group (group I) showed normal architecture of the islets of Langerhans. They appeared lightly stained, well defined with a regular outline and contained a large number of islet cells. The cells revealed vesicular nuclei and prominent nucleoli. The islets were surrounded by closely packed serous acini with a regular outline, rounded nuclei, and dark cytoplasm. Group II (cerulein-treated group) showed degenerative and necrotic changes in the form of a reduced dimension of the islet of Langerhans with an ill-defined border. Fewer islet cells were shown, compared to the control group, with pyknotic nuclei and intracytoplasmic vacuolations. Necrotic cells were found with many empty spaces indicating total cell necrosis. The islets were surrounded with distorted acini containing degenerated cells (Figure 2).

**Figure 3:**
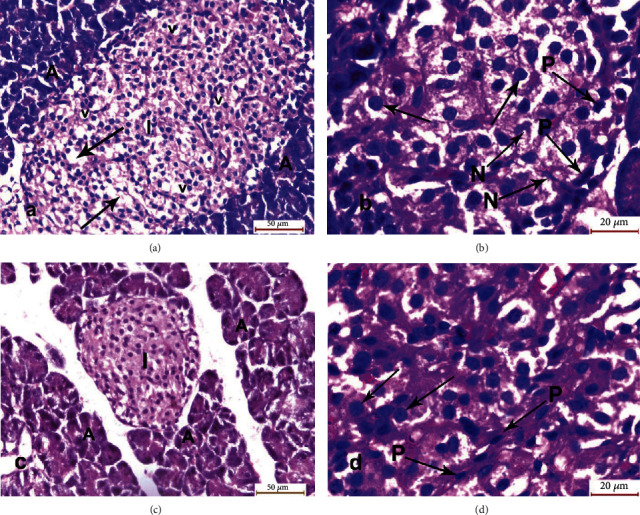
Photomicrographs of sections of the pancreatic specimens of the rats. (a) Group III showing lightly stained, well-defined islet of Langerhans (I) with some cellular intracytoplasmic vacuolations (v) and empty spaces (arrows) denoting cellular necrosis. Distorted acini with degenerated cells (A) appeared. (b) Higher magnification of group III showed islets cells having vesicular nuclei with prominent nucleoli (arrows) and some necrotic cells (N) with pyknotic nuclei (P). (c) Group IV showing lightly stained, well-defined islet of Langerhans (I) with regular outline containing normal islet cells and surrounded by closely packed normal serous acini (A). (d) Higher magnification of group IV showed islets cells having vesicular nuclei with prominent nucleoli (arrows) and few pyknotic nuclei (P) (H&E: a, c ×400; b, d ×1000).

The pancreatic specimens of group III (MSCs and cerulein-treated group) showed a lightly stained, well-defined islet of Langerhans with some cellular intracytoplasmic vacuolations and few necrotic cells. Distorted acini with degenerated cells also appeared. Many islet cells appeared normal with vesicular nuclei and prominent nucleoli, while some of them were necrotic with pyknotic nuclei. Group IV (MSCs, antioxidant mixture, and cerulein-treated group) revealed marked improvement of islets of Langerhans in the form of lightly stained, well-defined islets with a regular outline containing normal islet cells and surrounded by closely packed normal serous acini. The islet cells possessed vesicular nuclei, prominent nucleoli with few pyknotic nuclei (Figure 3).

#### 3.3.3. Immunohistochemical Reaction

**Figure 4:**
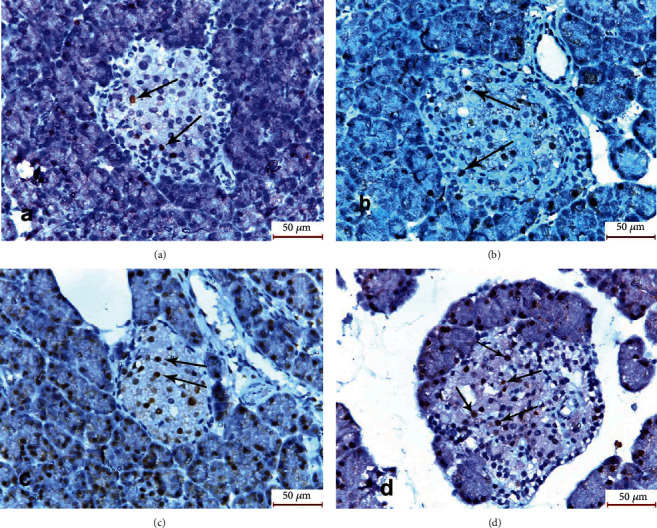
Photomicrographs of sections of the pancreatic specimens of the rats showing islets of Langerhans (I). (a) Group I showing few brown nuclei of islet cells (arrows). (b) Group II showing also few brown nuclei of islet cells (arrows). (c) Group III showing a moderate number of brown nuclei of islet cells (arrows). (d) Group IV showing larger number of brown nuclei of islet cells (arrows) (PCNA ×400).

The sections of the pancreatic specimens of the rats of the control group (group I) showed few PCNA reactions in the form of brown nuclei of islet cells. Group II (cerulein-treated group) revealed also few PCNA reactions. The pancreatic specimens of group III (MSCs and cerulein-treated group) revealed a moderate number of brown nuclei of islet cells. Group IV (MSCs, antioxidant mixture, and cerulein-treated group) showed a larger number of brown nuclei of islet cells (Figure 4).

**Figure 5:**
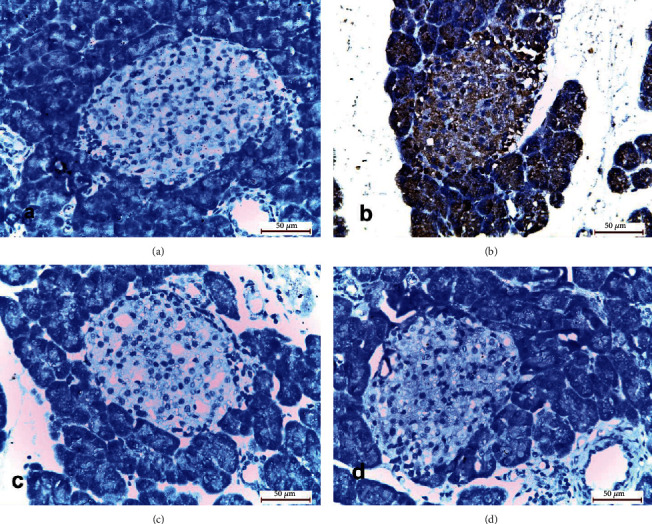
Photomicrographs of sections of the pancreatic specimens of the rats showing islets of Langerhans (I). (a) Group I, (c) group III, and (d) group IV showing absence of brown discoloration of islet cells. (b) Group II showing brown discoloration of islet cells and acini (arrows) (caspase-3 ×400).

Regarding the reaction to caspase-3, the islets of Langerhans of groups I, III, and IV showed absence of brown discoloration of islet cells denoting absence of caspase-3 reaction, while that of group II (cerulein-treated group) revealed brown discoloration of islet cells and acini (Figure 5).

The mean area percent of the positive immune reaction for PCNA in group III (cerulein+MSCs-treated group) was significantly increased compared with the control group and group II (cerulein-treated group). In addition, it was significantly increased in group IV (cerulein+antioxidants+MSCs-treated group) compared with the other three groups. The mean area percent of the positive immune reaction for caspase-3 in group II (cerulein-treated group) was significantly increased compared with the control group (group I) and was significantly decreased in group III (cerulein+MSCs-treated group) and group IV (cerulein+antioxidants+MSCs-treated group) compared with group II (cerulein-treated group) (Table 3).

## 4. Discussion

SAP is a life-threatening condition associated with high morbidity and fatality rate. Despite the therapeutic trials, till now, SAP has a poor prognosis and represents the fourteenth leading cause of death from digestive system disease [18]. In AP, the pancreatic acinar cell damage induces a cascade of premature activation of proenzymes, acute inflammation, autodigestion, necrosis, and loss of both endocrine and exocrine functions of the pancreas. In AP, the imbalance between the production of proinflammatory cytokines and the systemic anti-inflammatory response results in the systemic inflammatory reaction, multiple organ damage, and high mortality rate [1, 2, 48, 49].

As MSCs have an immunomodulatory function and high differentiation ability into many cell types, they developed extended traction in the treatment of various inflammatory, degenerative, and immune disorders [50–52]. Jiang et al. [21] have shown that BM-MSCs can differentiate into endothelium, ectoderm, and endoderm at the single-cell level [53]. Migration and homing of MSCs to different injured tissues were detected both in human and animal models [54]. An emerging novel concept has been postulated, under extensive investigation, to use the MSCs as a promising therapy in numerous gastrointestinal diseases including AP [55]. Previous studies have shown conflicting results about the therapeutic abilities of transplanted MSCs to the injured pancreas. Multiple studies recorded that MSCs can differentiate into islet *β* cells [56–58]. Although other studies could not prove if MSCs can differentiate into pancreatic exocrine *β* cells [18], the current work investigated the therapeutic efficacy of BM-MSCs and the adjuvant therapeutic effect of ascorbic acid and NAC antioxidants on experimentally cerulein-induced AP in albino rats. The regenerative effects of BM-MSCs, on the engrafted groups (group III, IV) of the present work, were detected by homing of BM-MSCs within the pancreatic tissue, while the added protective role of antioxidants, AA, and NAC (group IV) have been shown by increased density of homing and proliferation of BM-MSC masses inside the pancreatic tissue. Reduction of the severity of SAP in the BM-MSCs transplanted groups (groups III and IV) was indicated by the improvement of the clinical parameters; the significantly higher levels of serum insulin, higher body weight, and lower blood glucose levels, in addition to the significant downregulation of three of the most important tissue inflammatory cytokines: IL-1*β*, TNF-*α*, and NF-*κβ* compared with the untreated group (group II). Similarly, previous studies demonstrated decreased blood glucose, proinflammatory cytokines, increased body weight, and serum insulin in MSCs-treated AP [59]. Another study showed reduced IL-1*β* and TNF-*α* mRNA expressions after MSC transplantation in both the lung and the pancreas in induced SAP [18]. These results could provide evidence on the therapeutic abilities of the transplanted BM-MSCs on AP most likely through their immune-modulatory effects, by reduction of T-cell infiltration and increased recruitment of regulatory T-cells. Moreover, the antioxidant therapeutic activity of vitamin C and NAC was demonstrated in experimentally induced tissue damage in albino rats [60]. As demonstrated in the present study, the adjuvant therapeutic value of AA and NAC antioxidants to BM-MSCs was confirmed by detection of significant higher body weight, lower blood glucose levels, and lower NF-*κβ* expression levels in combined therapy-treated group (group IV) compared with the BM-MSCs-treated group (group III). In concordance with these findings, the combined treatment with ascorbic acid and N-acetylcysteine has been demonstrated to reduce the pancreatic and hepatic damage in induced AP through the restoration of antioxidant enzyme activities [34]. In another study, NAC has been reported to delay NF-*κβ* activation in induced AP. Accordingly, pancreatic acinar cells failed to produce TNF-*α* [61]. Downregulation of pancreatic IL-6 has also been previously detected in response to NAC treatment in cerulein-induced AP models [62].

The participation of BM-MSC in the reconstruction of the injured pancreatic tissue was demonstrated, in the current work in both groups III and IV. In group III (BM-MSCs-treated group), the pancreatic specimens showed well-defined normal islets of Langerhans with few necrotic cells and pyknotic nuclei, while group IV (combined BM-MSCs and antioxidant-treated group) revealed marked improvement of islets of Langerhans with normal islet cells. Previous studies have confirmed the regeneration capabilities of BM-MSCs on the injured pancreatic tissue through the protection of the integrity of acinar cells, promotion of pancreatic angiogenesis, significant lessening of inflammation, and inhibition of cellular apoptosis [46, 63, 64].

The histopathological evidence of the adjuvant therapeutic effect of combined NAC and AA on induced AP has been demonstrated to reduce the degree of acinar cell degeneration, pancreatic edema, inflammatory infiltration, and intracellular vacuolization in the rat model [34]. Therefore, it could be concluded that oxidative injury plays an important role in the pathogenesis of acute tissue necrosis. Antioxidant agents, such as AA and NAC, are capable of limiting the tissue damage produced during AP through the restoration of tissue antioxidant enzyme activities [65]. In the present study, two distinct diagnostic biomarkers were quantitatively measured by histomorphometric measurements to evaluate the response to various treatment lines used, the degree of PCNA expression as a biomarker for tissue regeneration [66, 67] and of caspase-3 expression as a biomarker of cellular apoptosis. The combined therapy of antioxidants and BM-MSCs showed very good prognostic indicators with a significantly high level of PCNA and low level of caspase-3 compared to the other groups of rat models. Caspases are essential components of mammalian apoptotic machines. Caspase-3 is a prototypical enzyme that is activated during apoptosis in a wide diversity of tissues [68].

## 5. Conclusion

From the present study, it could be concluded that pancreatic tissue regeneration in AP could be achieved by BM-MSC transplantation as a promising, efficient, and reliable treatment. However, adjuvant antioxidants such as NAC and AA combined therapy with MSCs showed more enhancement of cellular regeneration. TNF-*α*, IL 1*β*, and NF-*κβ* are helpful biomarkers for the assessment of MSC therapeutic efficiency. Therefore, further studies on BM-MSCs-based SAP therapy would be conducted for experimental evaluation and validation, to be translated into clinical practice.

## Figures and Tables

**Table 1:** Mean and standard deviation of body weight, fasting blood glucose, and fasting serum insulin among the studied groups.

Groups	I (control)	II (cerulein)	III (cerulein+MSCs)	IV (cerulein+antioxidants+MSCs)
Mean ± SD and percentage of the body weight (gm)	225.2 ± 14.13	98.2 ± 8.87^a^ 43.6%	150.4 ± 7.7^ab^ 66.79%	185.4 ± 8.56^abc^ 82.33%
Mean ± SD of the fasting blood glucose (mg/dl)	101.80 ± 9.37	286.2 ± 8.1^a^	147.4 ± 5.03^ab^	132.3 ± 5.72^abc^
Fasting serum insulin (*μ*U/ml)	3.04 ± 0.38	0.99 ± 0.31^a^	1.9 ± 0.34^ab^	2.52 ± 0.4^b^

^a^Significant to group I (*p* < 0.05); ^b^significant to group II (*p* < 0.05); ^c^significant to group III (*p* < 0.05).

**Table 2:** Mean and standard deviation of real-time PCR for IL-1*β*, TNF-*α*, and NF-*κβ* gene expressions among the studied groups.

Groups	I (control)	II (cerulein)	III (cerulein+MSCs)	IV (cerulein+antioxidants+MSCs)
IL-1*β*	0.60 ± 0.10	3.03 ± 0.13^a^	1.54 ± 0.22^ab^	1.31 ± 0.15^ab^
TNF-*α*	1.00 ± 0.16	201.49 ± 23.67^a^	8.99 ± 0.33^b^	5.69 ± 0.79^b^
NF-*κβ*	1.52 ± 0.46	819.90 ± 35.85^a^	318.74 ± 69.8^ab^	121.52 ± 23.62^abc^

^a^Significant to group I (*p* < 0.05); ^b^significant to group II (*p* < 0.05); ^c^significant to group III (*p* < 0.05).

**Table 3:** Mean and standard deviation of the area percent of the positive reaction of PCNA and caspase-3 among the studied groups.

Groups	I (control)	II (cerulein)	III (cerulein+MSCs)	IV (cerulein+antioxidants+MSCs)
PCNA	8.61 ± 0.69	5.22 ± 0.44	48.82 ± 5.25^ab^	81.40 ± 12.70^abc^
Caspase-3	0.04 ± 0.03	57.89 ± 5.50^a^	0.07 ± 0.04^b^	0.03 ± 0.02^b^

^a^Significant to group I (*p* < 0.05); ^b^significant to group II (*p* < 0.05); ^c^significant to group III (*p* < 0.05).

## Data Availability

The data used to support the findings of this study are included in the article.

## References

[1] Frossard J. L., Steer M. L., Pastor C. M. (2008). Acute pancreatitis. *Lancet*.

[2] Chan Y. C., Leung P. S. (2007). Acute pancreatitis: animal models and recent advances in basic research. *Pancreas*.

[3] McKay C. J., Imrie C. W. (2004). The continuing challenge of early mortality in acute pancreatitis. *The British Journal of Surgery*.

[4] de Waal Malefyt R., Abrams J., Bennett B., Figdor C. G., de Vries J. E. (1991). Interleukin 10(IL-10) inhibits cytokine synthesis by human monocytes: an autoregulatory role of IL-10 produced by monocytes. *The Journal of Experimental Medicine*.

[5] Zyromski N., Murr M. M. (2003). Evolving concepts in the pathophysiology of acute pancreatitis. *Surgery*.

[6] Ghosh S., May M. J., Kopp E. B. (1998). NF-*κ*B and Rel proteins: evolutionarily conserved mediators of immune responses. *Annual Review of Immunology*.

[7] Habib M. Z., Ebeid M. A., el Faramawy Y. (2020). Effects of lithium on cytokine neuro-inflammatory mediators, Wnt/*β*-catenin signaling and microglial activation in the hippocampus of chronic mild stress- exposed rats. *Toxicology and Applied Pharmacology*.

[8] Ramudo L., Manso M. A., Sevillano S., de Dios I. (2005). Kinetic study of TNF-*α* production and its regulatory mechanisms in acinar cells during acute pancreatitis induced by bile–pancreatic duct obstruction. *The Journal of Pathology*.

[9] Denham W., Yang J., Fink G. (1997). Gene targeting demonstrates additive detrimental effects of interleukin 1 and tumor necrosis factor during pancreatitis. *Gastroenterology*.

[10] Malleo G., Mazzon E., Siriwardena A. K., Cuzzocrea S. (2007). Role of tumor necrosis factor-*α* in acute pancreatitis: from biological basis to clinical evidence. *Shock*.

[11] Malleo G., Mazzon E., Genovese T. (2008). Effects of thalidomide in a mouse model of cerulein-induced acute pancreatitis. *Shock*.

[12] Abraham E., Wunderink R., Silverman H. (1995). Efficacy and safety of monoclonal antibody to human tumor necrosis factor *α* in patients with sepsis syndrome. A randomized, controlled, double-blind, multicenter clinical trial. *Journal of the American Medical Association*.

[13] Gukovskaya A. S., Mouria M., Gukovsky I. (2002). Ethanol metabolism and transcription factor activation in pancreatic acinar cells in rats. *Gastroenterology*.

[14] Shi C., Zhao X., Wang X., Andersson R. (2005). Role of nuclear factor-*κ*B, reactive oxygen species and cellular signaling in the early phase of acute pancreatitis. *Scandinavian Journal of Gastroenterology*.

[15] Altavilla D., Famulari C., Passaniti M. (2003). Lipid peroxidation inhibition reduces NF-*κ*B activation and attenuates cerulein-induced pancreatitis. *Free Radical Research*.

[16] Virlos I., Mazzon E., Serraino I. (2004). Calpain I inhibitor ameliorates the indices of disease severity in a murine model of cerulein-induced acute pancreatitis. *Intensive Care Medicine*.

[17] Steinle A. U., Weidenbach H., Wagner M., Adler G., Schmid R. M. (1999). NF-*κ*B/Rel activation in cerulein pancreatitis. *Gastroenterology*.

[18] Zhao H., He Z., Huang D. (2016). Infusion of bone marrow mesenchymal stem cells attenuates experimental severe acute pancreatitis in rats. *Stem Cells International*.

[19] Ung K. A., Rydberg L., Modin S., Kyleback A., Modin M. (2011). A preventive effect of unfractionated heparin on post-ERCP pancreatitis is suggested by positive effects on laboratory markers. *Hepato-Gastroenterology*.

[20] Feng D., Park O., Radaeva S. (2012). Interleukin-22 ameliorates cerulein-Induced pancreatitis in mice by inhibiting the autophagic pathway. *International Journal of Biological Sciences*.

[21] Jiang Y., Jahagirdar B. N., Reinhardt R. L. (2002). Pluripotency of mesenchymal stem cells derived from adult marrow. *Nature*.

[22] Takahashi K., Tanabe K., Ohnuki M. (2007). Induction of pluripotent stem cells from adult human fibroblasts by defined factors. *Cell*.

[23] Takahashi K., Yamanaka S. (2006). Induction of pluripotent stem cells from mouse embryonic and adult fibroblast cultures by defined factors. *Cell*.

[24] Tylicki L., Rutkowski B., Horl W. H. (2003). Antioxidants: a possible role in kidney protection. *Kidney & Blood Pressure Research*.

[25] Bulucu F., Vural A., Aydin A., Sayal A. (2000). Oxidative stress status in adults with nephrotic syndrome. *Clinical Nephrology*.

[26] Naidu A. (2003). Vitamin C in human health and disease is still a mystery? An overview. *Nutrition Journal*.

[27] Kehinde O. S., Christianah O. I., Oyetunji O. A. (2018). Ascorbic acid and sodium benzoate synergistically aggravates testicular dysfunction in adult Wistar rats. *International Journal of Physiology, Pathophysiology and Pharmacology*.

[28] Alhadlaq A., Mao J. J. (2004). Mesenchymal stem cells: isolation and therapeutics. *Stem Cells and Development*.

[29] El Sadik A. O., El Ghamrawy T. A., Abd El-Galil T. I. (2015). The effect of mesenchymal stem cells and chitosan gel on full thickness skin wound healing in albino rats: histological, immunohistochemical and fluorescent study. *PLoS One*.

[30] Ip J. K., Wu Y., Huang J., Zhang L., Pratt R. E., Dzau V. J. (2007). Mesenchymal stem cells use integrin *β*1 not CXC chemokine receptor 4 for myocardial migration and engraftment. *Molecular Biology of the Cell*.

[31] Ode A., Kopf J., Kurtz A. (2011). CD73 and CD29 concurrently mediate the mechanically induced decrease of migratory capacity of mesenchymal stromal cells. *European Cells & Materials*.

[32] Kyriakou C., Rabin N., Pizzey A., Nathwani A., Yong K. (2008). Factors that influence short-term homing of human bone marrow-derived mesenchymal stem cells in a xenogeneic animal model. *Haematologica*.

[33] Louis K. S., Siegel A. C. (2011). Cell viability analysis using trypan blue: manual and automated methods. *Methods in Molecular Biology*.

[34] Eşrefoğlu M., Gül M., Ateş B., Batçıoğlu K., Selimoğlu M. A. (2006). Antioxidative effect of melatonin, ascorbic acid and N-acetylcysteine on caerulein-induced pancreatitis and associated liver injury in rats. *World Journal of Gastroenterology*.

[35] Omar I. A., Aboulkhair G. A. (2017). Effect of bone marrow versus adipose tissue derived mesenchymal stem cells on the pancreas of streptozotocin-induced diabetes mellitus type I in adult male rats (histological study). *Egyptian Journal of Histology*.

[36] Ergul Y., Erkan T., Uzun H., Genc H., Altug T., Erginoz E. (2010). Effect of vitamin C on oxidative liver injury due to isoniazid in rats. *Pediatrics International*.

[37] Kannan G. M., Flora S. J. S. (2006). Combined administration of *N*-acetylcysteine and monoisoamyl DMSA on tissue oxidative stress during arsenic chelation therapy. *Biological Trace Element Research*.

[38] Selim S., Selim A. O. (2013). Effect of streptozotocin-induced diabetes mellitus on the cerebellar cortex of adult male albino rats. *The Egyptian Journal of Histology*.

[39] Kulkarni C. P., Bodhankar S. L., Ghule A. E., Mohan V., Thakurdesai P. A. (2012). Antidiabetic activity of Trigonella foenumgraecum L. seeds extract (ind01) in neonatal streptozotocin-induced (n-stz) rats. *Diabetologia Croatica*.

[40] Mani B. K., Uchida A., Lee Y. (2017). Hypoglycemic effect of combined ghrelin and glucagon receptor blockade. *Diabetes*.

[41] Wagner G. P., Kin K., Lynch V. J. (2012). Measurement of mRNA abundance using RNA-seq data: RPKM measure is inconsistent among samples. *Theory in Biosciences*.

[42] Cha J., Falanga V. (2007). Stem cells in cutaneous wound healing. *Clinics in Dermatology*.

[43] Porter A. G., Jänicke R. U. (1999). Emerging roles of caspase-3 in apoptosis. *Cell Death and Differentiation*.

[44] Zedan W., Mourad M. I., Abd El-Aziz S. M. (2015). Evaluation of caspase 3 as a target for apoptosis induced via chemotherapy in rats. *International Journal of Advanced Research*.

[45] Foley J. F., Dietrich D. R., Swenberg J. A., Maronpot R. R. (1991). Detection and evaluation of proliferating cell nuclear antigen (PCNA) in rat tissue by an improved immunohistochemical procedure. *Journal of Histotechnology*.

[46] Lilliu M. A., Solinas P., Cossu M. (2015). Diabetes causes morphological changes in human submandibular gland: a morphometric study. *Journal of Oral Pathology & Medicine*.

[47] Armitage P., Berry G. (1994). *Statistical Methods in Medical Research*.

[48] Chakraborty S., Kaur S., Muddana V. (2010). Elevated serum neutrophil gelatinase-associated lipocalin is an early predictor of severity and outcome in acute pancreatitis. *The American Journal of Gastroenterology*.

[49] Zhang Z., Wang Y., Dong M., Cui J., Rong D., Dong Q. (2012). Oxymatrine ameliorates l-arginine-induced acute pancreatitis in rats. *Inflammation*.

[50] Martin C. M., Hawke T. J., Garry D. J., Runge M. S., Patterson C. (2006). Stem cells and muscle regeneration. *Principles of Molecular Medicine*.

[51] Wang M., Tsai B. M., Crisostomo P. R., Meldrum D. R. (2006). Pre-treatment with adult progenitor cells improves recovery and decreases native myocardial proinflammatory signaling after ischemia. *Shock*.

[52] Urish K. L., Vella J. B., Okada M. (2009). Antioxidant levels represent a major determinant in the regenerative capacity of muscle stem cells. *Molecular Biology of the Cell*.

[53] Zhou Q., Law A. C., Rajagopal J., Anderson W. J., Gray P. A., Melton D. A. (2007). A multipotent progenitor domain guides pancreatic organogenesis. *Developmental Cell*.

[54] Sun X. C., Wu J. S., Wu J. M., Huang Z. M., Yu Z. (2013). Effects of intraperitoneal injection of marrow mesenchymal stem cells on intestinal barrier in acute pancreatitis. *Zhonghua Yi Xue Za Zhi*.

[55] Chela H., Romana B. S., Madabattula M. (2020). Stem cell therapy: a potential for the perils of pancreatitis. *The Turkish Journal of Gastroenterology*.

[56] Tang S. Q., Cao L. Z., Burkhardt B. R. (2004). In vivo and in vitro characterization of insulin-producing cells obtained from murine bone marrow. *Diabetes*.

[57] Oh S. H., Muzzonigro T. M., Bae S. H., LaPlante J. M., Hatch H. M., Petersen B. E. (2004). Adult bone marrow-derived cells trans-differentiating into insulin-producing cells for the treatment of type I diabetes. *Laboratory Investigation*.

[58] Karaoz E., Okcu A., Unal Z. S., Subasi C., Saglam O., Duruksu G. (2013). Adipose tissue-derived mesenchymal stromal cells efficiently differentiate into insulin-producing cells in pancreatic islet microenvironment both *in vitro* and *in vivo*. *Cytotherapy*.

[59] Xu Y., Chen J., Zhou H. (2020). Effects and mechanism of stem cells from human exfoliated deciduous teeth combined with hyperbaric oxygen therapy in type 2 diabetic rats. *Clinics*.

[60] Allam W., Mahmoud S., Mahmoud A. (2019). Protective effect of ascorbic acid and N- acetyl cysteine in aspartame induced nephrotoxicity in albino rats. *The Egyptian Journal of Forensic Sciences and Applied Toxicology*.

[61] Gukovsky I., Gukovskaya A. S., Blinman T. A., Zaninovic V., Pandol S. J. (1998). Early NF-*κ*B activation is associated with hormone-induced pancreatitis. *American Journal of Physiology-Gastrointestinal and Liver Physiology*.

[62] Ramudo L., Manso M. A. (2010). N-acetylcysteine in acute pancreatitis. *World Journal of Gastrointestinal Pharmacology and Therapeutics*.

[63] Jung K. H., Song S. U., Yi T. (2011). Human bone marrow-derived clonal mesenchymal stem cells inhibit inflammation and reduce acute pancreatitis in rats. *Gastroen-terology*.

[64] Gong J., Meng H. B., Hua J. (2014). The sdf-1/cxcr4 axis regulates migration of transplanted bone marrow mesenchymal stem cells towards the pancreas in rats with acute pancreatitis. *Molecular Medicine Reports*.

[65] Schmidt J., Rattner D. W., Lewandrowski K. (1992). A better model of acute pancreatitis for evaluating therapy. *Annals of Surgery*.

[66] Zhong W., Peng J., He H. (2008). Ki-67 and PCNA expression in prostate cancer and benign prostatic hyperplasia. *Clinical and Investigative Medicine*.

[67] Oltra B., Pozuelo J. M., Rodríguez R., Ingelmo I., Arriazu R., Santamaría L. (2014). Evaluation of PCNA, caspase 3 and E-cadherin on the ventral prostate of soy treated rats. *The Open Reproductive Science Journal*.

[68] Winter R. N., Kramer A., Borkowsky A., Kyprianou N. (2001). Loss of caspase-1 and caspase-3 protein expression in human prostate cancer. *Cancer Research*.

